# Incidental cardiovascular findings on chest CT scans requested for suspected COVID-19

**DOI:** 10.1590/1677-5449.210052

**Published:** 2022-01-07

**Authors:** José Maciel Caldas dos Reis, Glauco dos Santos Melo, Murilo Vasconcelos de Oliveira, Mariana Morgado Fernandez, Tereza Maria Meireles Fernandes da Silva, Hugo Luis da Silva Ferreira, Mariseth Carvalho de Andrade

**Affiliations:** 1 Centro Universitário Metropolitano da Amazônia – UNIFAMAZ, Belém, PA, Brasil.; 2 Hospital Santa Helena, São Paulo, SP, Brasil.; 3 Hospital Metropolitano de Urgência e Emergência, São Paulo, SP, Brasil.; 4 Hospital de Clínicas Gaspar Vianna – HCGV, Belém, PA, Brasil.; 5 Universidade do Estado do Pará – UEPA, Belém, PA, Brasil.; 6 Universidade Federal do Pará – UFPA, Belém, PA, Brasil.

**Keywords:** incidental finding, cardiovascular finding, tomography, COVID-19

## Abstract

**Background:**

Computed tomography scans of the chest are often requested as a complementary examination to investigate a clinical suspicion of pulmonary disease caused by the novel coronavirus 19 (COVID-19).

**Objectives:**

Our objective was to analyze the prevalence of incidental cardiovascular findings on chest CT scans requested to assess radiological signs suggestive of COVID-19 infection.

**Methods:**

This cross-sectional, descriptive, and retrospective study reviewed 1,444 chest tomographies conducted in the Radiology department of the Hospital de Clínicas Gaspar Vianna, from March 1 to July 30, 2020, describing the prevalence of images suggestive of viral pneumonia by COVID-19 and incidental pulmonary and cardiovascular findings.

**Results:**

The mean age of the patients was 50.6 ± 16.4 years and female sex was more frequent. Computed tomography without contrast was the most frequently used method (97.2%). Aortic and coronary wall calcification and cardiomegaly were the most prevalent cardiovascular findings. CT angiography revealed aortic aneurysms (9.7%), aortic dissection (7.3%) and thoracic aortic ulcers (2.4%).

**Conclusions:**

Incidental cardiovascular findings occurred in about half of the chest CT scans of patients with suspected COVID-19, especially aortic calcifications, cardiomegaly, and coronary calcification.

## INTRODUCTION

Incidental findings in imaging exams are defined as unexpected findings unrelated to the scope of the clinical indication.^1^^,^^2^ They are a common and well-known phenomenon in computed tomography (CT) examinations.^3^ However, the decisive factor is not the occurrence of a random finding, but its clinical relevance.^1^^,^^3^ In the literature on the subject, it is reported that incidental findings can be detected in up to 70% of all imaging investigations and, thankfully, the majority are of little clinical relevance.^1^^-^3

These findings can be classified according to their clinical importance, as major, moderate, or minor.^1^ From this perspective, newly-discovered tumors or aneurysms are of particular relevance, gallstones or pleural effusions can be considered moderately relevant, and uncomplicated liver cysts or simple vascular calcifications are irrelevant findings. The majority of incidental findings are clinically insignificant.^1^^,^^2^

Cardiovascular incidental findings include disorders such as aneurysms, calcifications of cardiac valves and arteries, and thromboembolisms, which can sometimes be clinically asymptomatic and require further investigation, or may need therapeutic interventions, as is the case with giant aortic aneurysms.^3^^-^5

Currently, because of the global health emergency faced by humanity and the scarcity of resources and limitations of large-scale confirmatory tests for the coronavirus 2019 disease (COVID-19), such as reverse transcription followed by polymerase chain reaction (RT-PCR),^6^ chest CT scans have come to play an important role supporting diagnosis because of their high sensitivity for detection of viral pneumonia and their potential to aid in assessment of disease progression and monitoring of treatment response.^7^^,^^8^

As a result, it has become necessary to increasing use imaging techniques with satisfactory accuracy and ensure availability of tomographic images in the majority of emergency units, since this is the preferred diagnostic examination in cases of suspected viral infection by COVID-19. Another advantage of CT, in addition to investigations related to the clinical complaint, is its capacity to identify incidental abnormalities, i.e., findings unrelated to the indications for requesting it.^6^^-^10

Considering the above, in the present study we intend to analyze the prevalence of incidental cardiovascular findings in chest CTs performed because of suspected COVID-19 at a tertiary hospital in the state of Pará, Brazil.

## METHOD

This is a retrospective and descriptive cross-sectional study conducted at the radiology service of the Hospital de Clínicas Gaspar Vianna (HCGV). The research corpus was collected at a general tertiary hospital specialized in cardiology, nephrology, and cardiovascular surgery, located in the city of Belém (PA).

This project was approved by the Research Ethics Committee at the institution and registered on the Plataforma Brasil (CAAE: 33706220.9.0000.0016) under ruling number 4.142.701.

Patients were identified using a computerized radiographic database that records all of the radiological studies performed by the radiology department. It was possible to include all chest CTs performed at the institution on patients from its various different sectors (wards, emergency department, or outpatients) from March 30 to July 2020 that had been requested because of indications related to respiratory symptoms, flu-like syndromes, or suspected COVID-19 pneumonia. CTs conducted because of other indications were excluded.

All CTs were performed using a Siemens Somatom Plus 16 multidetector scanner (Siemens Medical Systems Inc, Iselin, New Jersey, United States), and the tomographic images were recorded on a Vitria workstation and viewed with windows for bone (C800 W2000) and/or lung (C40 W80), both appropriate for the thorax. The examination was performed with the patient in dorsal decubitus with the following CT scan parameters: tube potential, 120 kVp; automatic tube current modulation, 30-70 mAs; pitch, 0.99-1.22 mm; matrix, 512 x 512; slice thickness, 1.0 mm; and field of view, 350 x 350 mm. Examinations reports were prepared by the institution’s three staff radiologists. When patients underwent more than one chest CT during the study period, only the results of the first examination were included in the analysis.

The research protocol comprised a list of 35 questions covering three principle topics: sociodemographic aspects, cardiovascular findings, and distribution of incidental findings.

Information was collected on the sample characteristics and organized in a spreadsheet in Microsoft^®^ Office Excel^®^ 2016.

The sample size was calculated based on an estimated prevalence of incidental cardiovascular findings of up to 60%, a standard error of 3.0%, and an alpha error of 5%, resulting in a minimum sample of 1,024 CT examinations.

Application of descriptive statistics involved drawing up tables and plotting graphs to present the results and calculating measures of position, such as arithmetic means and standard deviations.

The analytical statistics used to evaluate the results of variables for the sample were G tests and chi-square tests of fit for univariate tables. Descriptive and analytical statistics were computed using BioEstat^®^ 5.4 software. A significance level of α = 0.05, or 5%, was adopted for decision making, indicating significant results with an asterisk (*).

## RESULTS

During the period from March 1 through July 30, 2020, the HCGV radiology department performed a total of 1,444 chest CTs for suspected COVID-19 pulmonary infection.

Mean (standard deviation) patient age was 50.6±16.4 years. The youngest patient was 2 years old and the oldest was 99. There was a statistically significant difference (*p < 0.0001) between the proportions of age groups, with the greatest proportion of patients aged 40 to 49 years (25.7%).

There was a predominance of female patients (738; 51.1%), but the difference between sexes was not statistically significant (p = 0.4146).

The statistically significantly greatest proportion of requests for CT originated from the emergency department (48.8%), as shown in Table 1.

**Table 1:** Epidemiological profile of patients who underwent computed tomography of the chest for suspected COVID-19 at the Hospital de Clínicas Gaspar Vianna, from March to July 2020.

**Epidemiological variables**	**Frequency**	**% (n = 1,444)**	***p*-value**
**Sex**			0.4146
Female	738	51.1%	
Male	706	48.9%	
**Age group (years)**			< 0.0001^*^
< 30	145	10.0%	
30 to 39	244	16.9%	
40 to 49*	371	25.7%	
50 to 59	268	18.6%	
60 to 69	214	14.8%	
≥ 70	202	14.0%	
**Min / mean ± SD / max**	**2 / 50.6±16.4 / 99**		
**Scan requested by**			< 0.0001*
Emergency department*	704	48.8%	
External	409	28.3%	
Wards	290	20.1%	
Intensive care unit	41	2.8%	

* Chi-square test of fit.

COVID-19: coronavirus disease 2019; SD: standard deviation. Source: Radiology department electronic records.

The most common method used was CT without contrast (97.2%), used for a statistically significant proportion of the sample. Similarly, tomographic findings compatible with COVID-19 pulmonary involvement were observed in a significant 56% of the sample (*p = 0.0002) and the distribution pattern of pulmonary injury was < 25% ground-glass opacity in a statistically significant majority (*p < 0.0001) of patients, as shown in Table 2.

**Table 2:** Data from Chest CTs for suspected COVID-19 at the Hospital de Clínicas Gaspar Vianna, from March to July 2020.

**Chest CT**	**Frequency**	**% (n = 1,444)**	***p*-value**
**CT with contrast**			< 0.0001^*^
Yes	41	2.8%	
No*	1,403	97.2%	
**Compatible with COVID-19**			0.0002*
Yes*	809	56.0%	
No	635	44.0%	
**Ground-glass opacity**		**n = 809**	< 0.0001*
< 25%*	378	46.7%	
25 to 50%	239	29.5%	
> 50%	182	22.5%	
No information	10	1.2%	

* Chi-square test of fit.

COVID-19: coronavirus disease 2019; CT: computed tomography. Source: Radiology department electronic records.

Analysis of the tomographic findings compatible with COVID-19 pulmonary involvement revealed that a statistically significant percentage of patients had ground-glass opacity (56%). Additional compatible findings included consolidation (18.4%), pleural effusion (12.6%), and parenchymal bands (7.5%); tomographic characteristics suggestive of COVID-19, among the other findings identified in the CT reports, as shown in Table 3.

**Table 3:** Tomography findings compatible with COVID-19, from suspected COVID-19 cases at the Hospital de Clínicas Gaspar Vianna, March to July 2020.

**Tomographic characteristics**	**Frequency**	**% (n = 1,444)**	**Confidence interval**
Ground-glass opacity*	809	56.0%	53.5-58.6%
Consolidation	266	18.4%	16.4-20.4%
Pleural effusion	182	12.6%	10.9-14.3%
Parenchymal bands	109	7.5%	6.2-8.9%
Crazy-paving pattern	36	2.5%	1.7-3.3%
Air bronchogram	20	1.4%	0.8-2.0%
Reversed halo sign	7	0.5%	0.1-0.8%

* Chi-square test of fit.

COVID-19: coronavirus disease 2019. Source: Radiology department electronic records.

Incidental pulmonary findings were reported in 63.2% of chest CTs. The finding with the greatest proportion was pulmonary nodule (18.3%), which was statistically the most significant (*p < 0.0001). The most frequent other findings were peribronchial thickening (13.1%), band atelectasis (11.4%), atelectasis (9.5%), and emphysema (5.7%), as shown in Table 4.

**Table 4:** Incidental pulmonary findings on computed tomography of the chest in patients suspected of COVID-19, Hospital de Clínicas Gaspar Vianna, March to July 2020.

**Pulmonary findings**	**Frequency**	**% (n = 1,444)**	**Confidence interval**
Pulmonary nodule*	264	18.3%	16.3-20.3%
Peribronchial thickening	189	13.1%	11.3-14.8%
Band atelectasis	164	11.4%	9.7-13.0%
Atelectasis	137	9.5%	8.0-11.0%
Emphysema	83	5.7%	4.5-6.9%
Pleural thickening	49	3.4%	2.5-4.3%
Bronchiectasis	40	2.8%	1.9-3.6%
Residual calcification	25	1.7%	1.1-2.4%
Pulmonary cyst	24	1.7%	1.0-2.3%
Calcifed hilar lymph nodes	17	1.2%	0.6-1.7%
Other findings	21	1.5%	0.8-2.1%

* Chi-square test of fit.

COVID-19: coronavirus disease 2019. Source: Radiology department electronic records.

**Figure 1:**
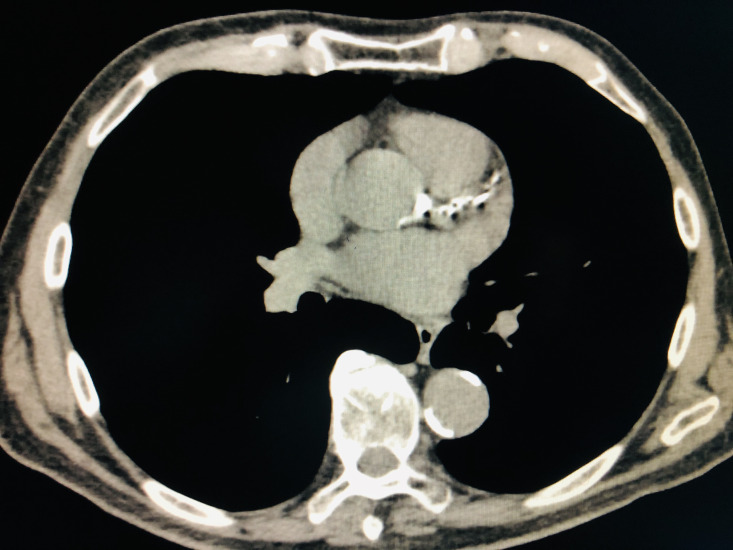
Incidental cardiovascular findings in computed tomography of the chest. Calcification of aortic and coronary wall.

**Figure 2:**
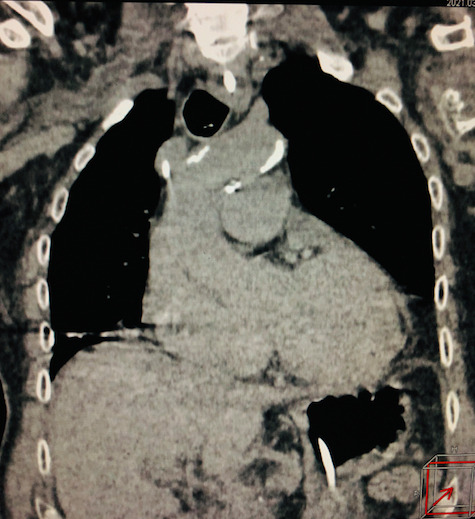
Incidental cardiovascular findings in computed tomography of the chest. Aortic wall calcification and cardiomegaly.

The database analysis was able to catalogue and distribute the cardiovascular findings present in 51.2% [confidence interval (CI) 48.7-53.8%] of the patients analyzed. The finding seen in the largest proportion of the sample was aortic wall calcification (21.8%), followed by cardiomegaly (10.5%), as illustrated in Table 5 and Figures 1 and 2. In turn, Table 6 shows the distribution of cardiovascular findings in the population over the age of 30 (1,299 patients). The most prevalent finding identified was aortic wall calcification (16.2%), followed by cardiomegaly (10.7%), and coronary calcification (4.5%).

**Table 5:** Incidental cardiovascular findings on computed tomography of the chest in patients suspected of COVID-19, Hospital de Clínicas Gaspar Vianna, March to July 2020.

**Cardiac findings**	**Frequency**	**% (n = 1,444)**	**Confidence interval**
Aortic wall calcification*	315	21.8%	19.7-23.9%
Cardiomegaly	152	10.5%	8.9-12.1%
Coronary calcification	72	5.0%	3.9-6.1%
Hemorrhagic pericarditis	50	3.5%	2.5-4.4%
Aortic root > 3 cm	35	2.4%	1.6-3.2%
Thoracic aorta elongation	32	2.2%	1.5-3.0%
Pulmonary artery trunk dilatation	26	1.8%	1.1-2.5%
Aneurysm	12	0.8%	0.4-1.3%
Pulmonary thromboembolism	10	0.7%	0.3-1.1%
Other findings	24	1.7%	1.0-2.3%

* Actively sought in hospitalized patients with clinical suspicion. p < 0.0001; chi-square test of fit. COVID-19: coronavirus disease 2019. Source: Radiology department electronic records.

**Table 6:** Incidental cardiovascular findings on computed tomography of the chest in patients over the age of 30 suspected of COVID-19, Hospital de Clínicas Gaspar Vianna, March to July 2020.

**Cardiac findings in adults**	**Frequency**	**% (n = 1,299)**	**Confidence interval**
Aortic wall calcification*	210	16.2%	13.7-28.7%
Cardiomegaly	139	10.7%	8.2-13.2%
Coronary calcification	58	4.5%	2.0-7.0%
Hemorrhagic pericarditis	44	3.4%	0.9-5.9%
Aortic root > 3 cm	31	2.4%	0.0-4.9%
Thoracic aorta elongation	23	1.8%	0.0-4.3%
Pulmonary artery trunk dilatation	16	1.2%	0.0-3.7%
Aneurysm	2	0.2%	0.0-2.7%

* Chi-square test of fit.

COVID-19: coronavirus disease 2019. Source: radiology department electronic records.

With regard to aortic conditions, there were 12 incidental diagnoses of thoracic aorta aneurysm (0.83%), seven in the ascending aorta and five in the thoracic descending aorta. These 12 aneurysms were diagnosed in 10 patients (two male patients had two aneurysms each, in the ascending and descending aorta).

**Figure 3:**
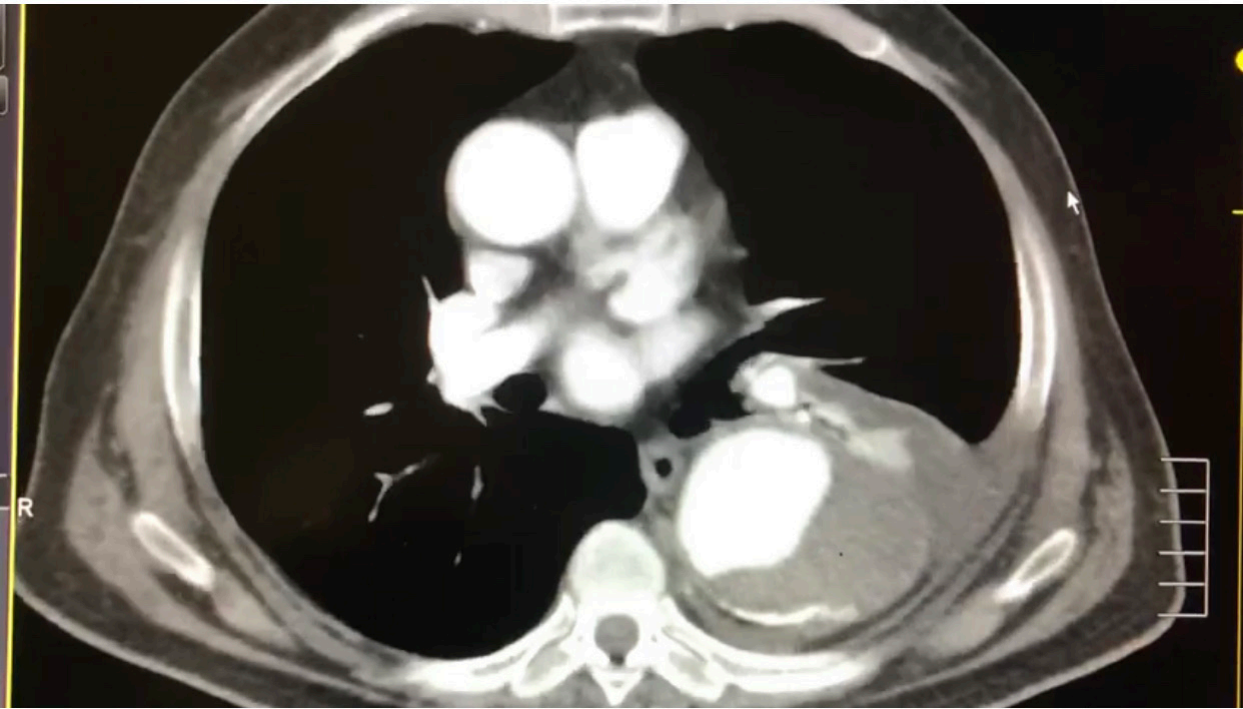
Incidental cardiovascular findings in computed tomography of the chest with contrast. Descending thoracic aortic aneurysm.

**Figure 4:**
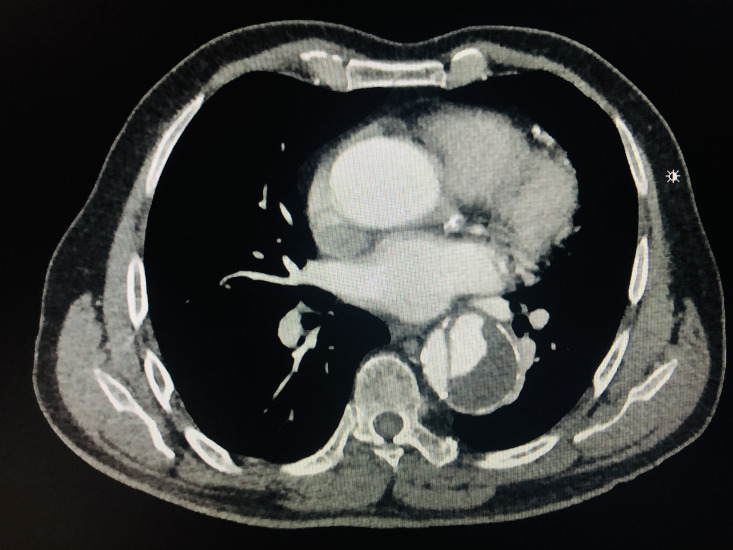
Incidental cardiovascular findings in computed tomography of the chest with contrast. Descending thoracic aorta dissection.

CT with contrast was conducted for 41 patients (2.8% of all scans), and yielded alternative diagnoses of aortic dissection in 7.3% (three of 41 scans) and thoracic aortic aneurysm in 9.7% (four of 41 scans). There were also findings of aortic ulcers in 2.4% (two of 41 scans). Figures 3 and 4 show examples of incidental aortic conditions detected with chest CT.

The prevalence of pulmonary thromboembolism (PTE) detected with computed tomography angiographies of the chest was 24.4% (10 of 41 examinations with contrast). Twenty-eight of these scans were of patients already in hospital and 13 were of patients referred from the emergency department. The incidental PTE findings were in two cases from the emergency department and eight of those already in hospital.

## DISCUSSION

The important role played by diagnostic imaging has increased the number of CT examinations performed and, as CT became more widely used, radiologists began to observe a wave of findings unrelated to the clinical indications for which the examinations were ordered.^1^^,^^3^^,^^4^

Confirmation of a COVID-19 diagnosis is based on RT-PCR of nasal or oropharyngeal samples collected with swabs. Patients infected by the SARS-Cov2 virus may present with changes seen on CT that are typical of the disease, such as ground-glass opacities. However, according to the Brazilian College of Radiology’s recommendations, CT should only be performed on symptomatic patients who have been admitted to hospital.^11^

Incidental findings are a well-known and common phenomenon in imaging exams.^1^^,^^4^^,^^7^^,^^12^ They can be classified according to their clinical importance, as major, moderate, or minor.^12^ Major findings include lesions suspected to be malignant diseases, such as thyroid nodules, changes to the thickness of the intestinal wall, and solid pancreatic or renal masses.^12^ Moderate findings, i.e. findings possibly of clinical relevance, include gallstones and pleural fluid accumulations or pleural effusion. Minor findings, or findings without clinical relevance, include simple renal or hepatic cysts, degenerative changes to the spinal column, and calcification of vessels. Many incidental findings are clinically insignificant.^1^^,^^2^^,^^4^^,^^7^

This study found that female patients predominated and the most frequent source of patients was the institution’s emergency department, which requested 48.8% of the examinations.

The routine chest CT protocol used at the HCGV is a low-dose, 1.0 mm slice program without intravenous contrast. The great majority of chest CTs (97.2%) were performed without contrast, with contrast reserved for cases in which PTE or other vascular disorders requiring more detailed analysis were suspected.

The most common source of Chest CTs requests was the emergency department (48.8%). This elevated demand from external patients (rather than patients already admitted to the wards or intensive care unit) is probably because of the hospital’s characteristics, since it has an open-door emergency service for cardiovascular and nephrology patients.

Although CT is far from being the only examination of choice recommended for diagnosis by the various different medical specialty societies, it has become a valuable tool to support diagnosis in these patients, in addition to its utility for monitoring progress and detecting possible complications.^12^^,^^13^ Thus, 56% of chest CTs requested had findings compatible with COVID-19 pulmonary involvement. The most characteristic findings were ground-glass opacity and consolidation; but many other findings can suggest pulmonary damage, depending on the disease phase and time since onset.^13^

A total of 63.2% of the CTs requested for diagnosis of presumed COVID-19 had incidental findings and the majority of these comprised pulmonary nodules (18.3%), peribronchial thickening (13.1%), band atelectasis (11.4%), and atelectasis (9.5%). Nodules were statistically significantly more common than the other pulmonary findings (p < 0.0001). It is believed that small nodules are not clinically relevant. However, eight patients exhibited findings of pulmonary masses larger than 3 cm (0.5% of the sample). This supports the hypothesis that the great majority of incidental findings are irrelevant, but the method can also detect asymptomatic injuries that need further management.^11^ Thus, although bilateral ground-glass opacity and consolidation are described as the predominant findings characteristic of imaging exams conducted for COVID-19, the manifestations seen on chest CT can vary from patient to patient and at different disease stages.^11^

Incidental cardiovascular findings were found in 51.2% (CI 48.7-53.8%) of the CT scans conducted and more than 40% had more than one finding. Calcification of aortic and coronary walls and cardiomegaly were the most common in the radiological reports. Less common findings involved hemorrhagic pericarditis and dilatation of the aortic root. These findings can be explained by the epidemiological profile observed in the study, since approximately 30% of the patients were over the age of 60, and also by the profile of the hospital’s patients in general, since it specializes in cardiovascular conditions and nephrology.

In an attempt to form more homogenous groups, a subset analysis was conducted after excluding data from 145 examinations of individuals less than 30 years old, leaving a subset of 1,299 CTs and revealing a similar distribution, with predominance of coronary and aorta wall calcification, cardiomegaly, and hemorrhagic pericarditis. Thus, regardless of its incidental nature early detection of potentially clinically relevant diseases, such as coronary calcification and aortic aneurysms or dissections, can change the prognosis of this population and have a positive impact on reduction of mortality and increase of quality of life.

Surov et al.^2^ showed that cardiovascular findings can be identified in 6.8% of patients with malignant diseases investigated with CT. In contrast, Jacobs et al.^4^ reported that in their study the frequency of aortic aneurysm varied from 0.07% to 3.4%,^4^ while the frequency of aortic dissection varied from 0.06% to 0.2%.

Although incidental cardiac findings may not be relevant to the immediate clinical management of patients with suspected COVID-19 pulmonary involvement, they can influence long-term clinical management and improve prognosis. For example, coronary and aorta wall calcifications are known markers of atherosclerosis and signs of underlying cardiovascular disease, very often subclinical, which can influence primary prevention of atherosclerotic events.^14^^-^17 On the other hand, cardiomegaly constitutes a late characteristic of left ventricular dysfunction and heart failure, both with poor prognosis, and could trigger further investigation and more effective cardiac treatment.^14^

Several different studies have shown that atherosclerotic plaques, calcifications, and aorta wall irregularities are very prevalent among patients with cardiovascular disease.^15^^-^17 Others have demonstrated that calcification of the descending aorta is related to calcification of the coronary arteries, an important predictor of cardiovascular disease. However, to date, no follow-up studies have been conducted to investigate the prognostic value of these abnormalities.^16^^,^^17^

Sverzellati et al.^18^ reported that 50% of 286 CT examinations ordered for pulmonary fibrosis, suspected pulmonary embolism, or lung cancer staging revealed potentially significant cardiovascular findings. Along the same lines, Choy et al.^19^ demonstrated that 61% of a consecutive series of routine chest CTs exhibited cardiac findings that merited reporting.

Previous studies of COVID-19 have shown that 63 to 67% of patients who died had cardiovascular comorbidities, most commonly hypertension, diabetes, and coronary cardiac disease,^20^^-^22 which are all factors that can be linked to vascular diseases.^19^^-^22

Aortic conditions were detected incidentally on chest CT in up to 2.4% of cases, among which aneurysms were the most prevalent abnormality, especially in CTs without contrast. In the absence of intravenous contrast, significant aortic dissections and ulcerations are generally undetectable, which can be considered a limitation of the method.^4^ In the present study, just 2.8% of the CT scans were conducted with contrast. Aneurysms and dissections were observed in 9.7% and 7.3% respectively and suggested in the radiologist’s report in 1.2% of scans without intravenous contrast. On the other hand, PTE was observed in 24.4% of the patients who had scans with contrast, constituting a significant sample of cases admitted to the intensive care unit, where the more severe COVID-19 cases predominate.

Thus, is it is important to emphasize that incidental findings constitute an important event for patient clinical outcomes, since a considerable proportion of them have comorbidities concomitant to the novel coronavirus infection and, consequently, are more susceptible to the disease’s complications. If the incidental diagnosis is made early and is of a relevant nature, it increases the likelihood of management and prompt treatment and, consequently, of better prognosis for the infected population.

The present study is subject to limitations, since it is a retrospective study with selection bias, because it analyzed a sample of outpatients and inpatients from a specialist cardiovascular service and the data collection period was relatively short. The CT reports were prepared by three different radiologists and there was no analysis by race or other subsets. Some cardiovascular findings were observed in scans with contrast, including ulcers and dissections, which could indicate that CTs without contrast are limited in this respect.

No analysis was conducted of correlations between cardiovascular findings and risk factors because the study is based on CT scans and reports that were requested because of diagnosis suggestive of COVID-19. Additional studies are ongoing at the institution with research protocols that include better stratification of outpatients and inpatients in order to obtain more trustworthy and explanatory results.

## CONCLUSIONS

Incidental cardiovascular findings were observed in approximately half of the chest CTs of patients with suspected COVID-19; more specifically, aortic calcifications, cardiomegaly, and coronary calcification.
