# Supplementary material for: Incidental cardiovascular findings on chest CT scans requested for suspected COVID-19
Source: J Vasc Bras. 2022 Jan 7;20:e20210052. doi: 10.1590/1677-5449.210052 (PMC8759582; doi:10.1590/1677-5449.210052)
Supplement: Translation

# Translation: Achados cardiovasculares incidentais em tomografias de tórax solicitadas para suspeita de COVID-19

**Authors:** José Maciel Caldas dos Reis, Glauco dos Santos Melo, Murilo Vasconcelos de Oliveira, Mariana Morgado Fernandez, Tereza Maria Meireles Fernandes da Silva, Hugo Luis da Silva Ferreira, Mariseth Carvalho de Andrade

**Affiliations:** 1 Centro Universitário Metropolitano da Amazônia – UNIFAMAZ, Belém, PA, Brasil.; 2 Hospital Santa Helena, São Paulo, SP, Brasil.; 3 Hospital Metropolitano de Urgência e Emergência, São Paulo, SP, Brasil.; 4 Hospital de Clínicas Gaspar Vianna – HCGV, Belém, PA, Brasil.; 5 Universidade do Estado do Pará – UEPA, Belém, PA, Brasil.; 6 Universidade Federal do Pará – UFPA, Belém, PA, Brasil.

**Keywords:** achados incidentais, achado cardiovascular, tomografia, COVID-19

## Abstract

**Contexto:**

As tomografias de tórax são frequentemente solicitadas como exames complementares para avaliação de suspeita clínica de afecção pulmonar pelo novo coronavírus 19 (COVID-19).

**Objetivos:**

Nosso objetivo foi analisar a prevalência dos achados cardiovasculares incidentais em tomografias de tórax solicitadas para avaliar sinais radiológicos sugestivos de COVID-19.

**Métodos:**

Por meio de um estudo transversal, descritivo e retrospectivo, foram revisadas 1.444 tomografias de tórax realizadas no setor de radiologia do Hospital de Clínicas Gaspar Vianna, no período de 1° de março a 30 de julho de 2020, com a descrição da prevalência de imagens sugestivas de pneumonia viral pelo COVID-19, além de achados pulmonares e cardiovasculares incidentais.

**Resultados:**

A média de idade dos pacientes foi 50,6±16,4 anos, sendo o sexo feminino o mais frequente. A tomografia sem contraste foi o método mais utilizado (97,2%), e opacidades em vidro fosco foram identificadas em 56,0% dos casos. Achados incidentais cardiovasculares ocorreram em 51,2% (intervalo de confiança 48,7%-53,8%) das tomografias, prevalecendo calcificação da parede aórtica (21,8%), cardiomegalia (10,5%), e calcificação coronária (5,0%). Nas tomografias com contraste, evidenciaram-se aneurismas de aorta (9,7%), dissecção de aorta (7,3%) e úlceras de aorta torácica (2,4%).

**Conclusões:**

Achados cardiovasculares incidentais ocorreram em aproximadamente metade das tomografias de tórax de pacientes com suspeita de COVID-19, mais especificamente, calcificações da parede da aorta, cardiomegalia e calcificação coronária.

## INTRODUÇÃO

Achados incidentais em exames de imagem são definidos como achados inesperados, não relacionados ao escopo da indicação clínica^1^^,^^2^. São fenômenos conhecidos e comuns em exames de tomografia computadorizada (TC)^3^. No entanto, o fator decisivo não é a ocorrência de um achado aleatório, mas a sua relevância clínica^1^^,^^3^. Na literatura pesquisada a respeito, observa-se que podem achados incidentais ser detectados em até 70% de todas as investigações por imagem e, felizmente, a maioria tem baixa relevância clínica^1^^-^3.

Esses achados podem ser classificados, de acordo com sua importância clínica, em maiores, moderados e menores^1^. Assim, as neoplasias recém-descobertas ou aneurismas são particularmente relevantes, cálculos biliares ou derrames pleurais podem ser considerados moderadamente relevantes, enquanto que cistos não complicados de fígado ou rim e calcificações vasculares simples são achados irrelevantes. A maioria dos achados incidentais são clinicamente não significativos^1^^,^^2^.

Os achados incidentais cardiovasculares compreendem distúrbios como aneurismas, calcificações das válvulas e artérias cardíacas, e tromboembolismos, que às vezes podem ser clinicamente assintomáticos e exigir mais investigação ou necessitar de intervenções terapêuticas, como é o caso de aneurismas aórticos gigantes^3^^-^5.

Atualmente, devido à emergência de saúde global enfrentada pela humanidade e à escassez de recursos e limitações de exames confirmatórios em larga escala para a doença do coronavírus 2019 (*coronavirus disease*, COVID-19), como a transcrição reversa seguida de reação em cadeia da polimerase (*reverse transcription-polymerase chain reaction*, RT-PCR)^6^, a TC de tórax passou a exercer importante função no auxílio diagnóstico, em virtude da sua alta sensibilidade na detecção de pneumonia viral, podendo colaborar potencialmente na avaliação de progressão da doença e no monitoramento da resposta à terapia^7^^,^^8^.

Dessa forma, o aumento do uso de técnicas de imagem com acurácia satisfatória e a frequente disponibilidade das imagens tomográficas para o exame diagnóstico preferencial em caso de suspeita de infeção viral pelo COVID-19, na maioria das unidades de emergência, tem sido uma prática necessária. Outra vantagem da TC, além da investigação relacionada à queixa clínica, é a capacidade de identificar anormalidades incidentais, ou seja, achados não relacionados à indicação do estudo^6^^-^10.

Considerando essa discussão, com o presente estudo pretendemos avaliar a prevalência de achados cardiovasculares incidentais em TCs de tórax realizadas por suspeitas de COVID-19 em um hospital de referência no Pará.

## MÉTODO

Trata-se de um estudo transversal, retrospectivo e descritivo, realizado no serviço de radiologia do Hospital de Clínicas Gaspar Vianna (HCGV). O levantamento do *corpus* de pesquisa foi realizado em hospital geral terciário com perfil clínico cardiológico e nefrológico e cirúrgico cardiovascular, na cidade de Belém (PA).

Este trabalho foi aprovado pelo Comitê de Ética em Pesquisa da instituição e registrado na Plataforma Brasil (CAAE: 33706220.9.0000.0016), sob o parecer número 4.142.701.

Os pacientes foram identificados por meio de um banco de dados radiográfico computadorizado que registra todos os estudos radiológicos realizados pelo departamento de radiologia. Foi possível incluir no estudo todas as TCs de tórax realizadas na instituição, oriundas dos diversos setores (unidades de internamento, pronto atendimento, ou de pacientes ambulatoriais), no período de 1° de março a 30 de julho de 2020, cuja indicação tenha relação com sintomas respiratórios da síndrome gripal ou suspeita de pneumonia por COVID-19. Foram excluídas TCs realizadas por outras indicações.

As TCs foram obtidas com um *scanner* multidetector Siemens Somatom Plus 16 (Siemens Medical Systems Inc, Iselin, Nova Jérsei, EUA), e os cortes tomográficos gravados em uma estação de trabalho (*workstation* - Vitria), visualizados com janelas para osso (C800 W2000) e/ou janela para pulmão (C40 W80), ambas adequadas à avaliação de tórax. Para a realização do exame, o paciente era posicionado em decúbito dosal, e os principais parâmetros de varredura foram os seguintes: tensão do tubo, 120 kVp; modulação automática da corrente do tubo, 30-70 mAs; passo, 0,99-1,22 mm; matriz, 512 × 512; espessura do corte, 1,0 mm; e campo de visão, 350 × 350 mm. Os exames foram laudados pelos três radiologistas titulados da instituição. Quando os indivíduos do estudo submeteram-se a mais de uma TC de tórax durante o acompanhamento, foi considerado apenas o resultado do primeiro exame.

O protocolo da pesquisa consistiu em 35 perguntas que envolveram três tópicos principais: aspectos sociodemográficos, achados cardiovasculares e distribuição dos achados incidentais.

As informações da caracterização amostral foram apuradas e sistematizadas em planilha elaborada no *software* Microsoft^®^ Office Excel^®^ 2016.

O dimensionamento amostral foi baseado na estimativa de uma prevalência de até 60% de achados cardiovasculares incidentais, erro padrão de 3,0% e nível alfa de 5%, resultando em uma amostra mínima de 1.024 exames.

Na aplicação da estatística descritiva, foram construídos tabelas e gráficos para apresentação dos resultados, e calculadas as medidas de posição, como média aritmética e desvio padrão.

A estatística analítica utilizada para avaliar os resultados das variáveis da amostra ocorreu por meio dos testes G e qui-quadrado de aderência para tabelas univariadas. As estatísticas descritiva e analítica foram realizadas pelo *software* BioEstat^®^ 5.4. Para a tomada de decisão, adotou-se o nível de significância α = 0,05, ou 5%, sinalizando com asterisco (*) os valores significantes.

## RESULTADOS

No período de 1° de março a 30 de julho de 2020, foram realizadas 1.444 TCs de tórax no setor de radiologia do HCGV por suspeita de acometimento pulmonar por COVID-19.

A média (desvio padrão) de idade dos pacientes foi de 50,6±16,4 anos. O paciente mais jovem tinha 2 anos, e o de maior idade tinha 99 anos. Houve diferença estatisticamente significante (*p < 0,0001) entre as faixas etárias dos pesquisados, com maior proporção de pacientes entre 40 a 49 anos (25,7%).

Houve predominância do sexo feminino (738; 51,1%). No entanto, não houve diferença estatisticamente significante (p = 0,4146) entre os sexos.

A parte estatisticamente mais significativa das solicitações para realização do exame foi provenientes do pronto atendimento (48,8%), como mostra a Tabela 1.

**Tabela 1:** Perfil epidemiológico dos pacientes submetidos a tomografia computadorizada de tórax por suspeita de COVID-19, Hospital de Clínicas Gaspar Vianna, março a julho de 2020.

**Perfil epidemiológico**	**Frequência**	**% (n = 1.444)**	**p-valor**
**Sexo**			0,4146
Feminino	738	51,1%	
Masculino	706	48,9%	
**Faixa etária (anos)**			< 0,0001*
< 30	145	10,0%	
30 a 39	244	16,9%	
40 a 49*	371	25,7%	
50 a 59	268	18,6%	
60 a 69	214	14,8%	
≥ 70	202	14,0%	
**Mín / média ± DP / máx**	**2 / 50,6±16,4 / 99**		
**Origem da solicitação**			< 0,0001*
Pronto atendimento*	704	48,8%	
Externo	409	28,3%	
Internado	290	20,1%	
Unidade de terapia intensiva	41	2,8%	

* Teste qui-quadrado de aderência.

COVID-19: doença do coronavírus 2019 (*coronavirus disease* 2019); DP: desvio padrão. Fonte: Sistema eletrônico do setor de radiologia.

A TC sem contraste foi o método mais comum (97,2%), realizado em proporção estatisticamente significante da amostra. Da mesma forma, achados tomográficos compatíveis com acometimento pulmonar pela COVID-19 ocorreram de forma significante (*p = 0,0002) em 56% da amostra, e a maioria estatisticamente significante (*p < 0,0001) apresentou opacidade em vidro fosco < 25% como padrão de distribuição da lesão pulmonar, conforme Tabela 2.

**Tabela 2:** Dados das TCs de tórax realizadas em pacientes suspeitos de COVID-19, Hospital de Clínicas Gaspar Vianna, março a julho de 2020.

**TC de tórax**	**Frequência**	**% (n = 1.444)**	**p-valor**
**TC com contraste**			< 0,0001*
Sim	41	2,8%	
Não*	1.403	97,2%	
**Compatível com COVID-19**			0,0002*
Sim*	809	56,0%	
Não	635	44,0%	
**Opacidade em vidro fosco**		**n = 809**	< 0,0001*
< 25%*	378	46,7%	
25 a 50%	239	29,5%	
> 50%	182	22,5%	
Sem informação	10	1,2%	

* Teste qui-quadrado de aderência.

COVID-19: doença do coronavírus 2019 (*coronavirus disease* 2019); TC: tomografia computadorizada. Fonte: Sistema eletrônico do setor de radiologia.

A análise dos achados tomográficos compatíveis com comprometimento pulmonar pela COVID-19 evidenciou um percentual estatisticamente significante de pacientes com opacidade em vidro fosco (56%). Achados adicionais compatíveis incluíram consolidação (18,4%), derrame pleural (12,6%) e bandas parenquimatosas (7,5%), características tomográficas sugestivas de COVID-19, entre outras identificadas nos laudos, conforme Tabela 3.

**Tabela 3:** Achados tomográficos compatíveis com COVID-19, obtidos em casos suspeitos no Hospital de Clínicas Gaspar Vianna, março a julho de 2020.

**Características tomográficas**	**Frequência**	**% (n = 1.444)**	**Intervalo de confiança**
Opacidade em vidro fosco*	809	56,0%	53,5-58,6%
Consolidação	266	18,4%	16,4-20,4%
Derrame pleural	182	12,6%	10,9-14,3%
Bandas parenquimatosas	109	7,5%	6,2-8,9%
Pavimentação em mosaico	36	2,5%	1,7-3,3%
Brancograma aéreo	20	1,4%	0,8-2,0%
Sinal do halo invertido	7	0,5%	0,1-0,8%

* p < 0,0001; teste qui-quadrado de aderência.

COVID-19: doença do coronavírus 2019 (*coronavirus disease* 2019). Fonte: Sistema eletrônico do setor de radiologia.

Achados pulmonares incidentais foram evidenciados em 63,2% das TCs de tórax, sendo o nódulo pulmonar o de maior proporção (18,3%) e estatisticamente mais significativo (*p < 0,0001) entre os demais. Achados adicionais incluíram espessamento peribrônquico (13,1%), faixas fibroatelectásicas (11,4%), atelectasia (9,5%) e enfisema (5,7%) como os mais frequentes, como evidencia a Tabela 4.

**Tabela 4:** Achados pulmonares incidentais em tomografias computadorizada de tórax de pacientes suspeitos de COVID-19, Hospital de Clínicas Gaspar Vianna, março a julho de 2020.

**Achados pulmonares**	**Frequência**	**% (n = 1.444)**	**Intervalo de confiança**
Nódulo pulmonar*	264	18,3%	16,3-20,3%
Espessamento peribrônquico	189	13,1%	11,3-14,8%
Faixas fibroatelectásicas	164	11,4%	9,7-13,0%
Atelectasia	137	9,5%	8,0-11,0%
Enfisema	83	5,7%	4,5-6,9%
Espessamento pleural	49	3,4%	2,5-4,3%
Bronquiectasias	40	2,8%	1,9-3,6%
Calcificação residual	25	1,7%	1,1-2,4%
Cisto pulmonar	24	1,7%	1,0-2,3%
Linfonodos hilares calcificados	17	1,2%	0,6-1,7%
Outros achados	21	1,5%	0,8-2,1%

* p < 0,0001; teste qui-quadrado de aderência.

COVID-19: doença do coronavírus 2019 (*coronavirus disease* 2019). Fonte: Sistema eletrônico do setor de radiologia.

Em análise dos bancos de dados, foi possível catalogar e distribuir os achados cardiovasculares presentes em 51,2% [intervalo de confiança (IC) 48,7-53,8%] dos pacientes analisados. O achado de maior proporção na amostra foi a calcificação da parede aórtica (21,8%), seguida da cardiomegalia (10,5%), conforme ilustram a Tabela 5 e as Figuras 1 e 2. Já na Tabela 6, observa-se a distribuição dos achados cardiovasculares na população com mais de 30 anos de idade (1.299 pacientes). A identificação predominante foi calcificação da parede aórtica (16,2%), seguida de cardiomegalia (10,7%) e calcificação coronária (4,5%).

**Tabela 5:** Achados cardiovasculares incidentais em tomografias computadorizadas de tórax de pacientes suspeitos de COVID-19, Hospital de Clínicas Gaspar Vianna, março a julho de 2020.

**Achados cardíacos**	**Frequência**	**% (n = 1.444)**	**Intervalo de confiança**
Calcificação da parede aórtica*	315	21,8%	19,7-23,9%
Cardiomegalia	152	10,5%	8,9-12,1%
Calcificação coronária	72	5,0%	3,9-6,1%
Derrame pericárdio	50	3,5%	2,5-4,4%
Raiz aórtica > 3 cm	35	2,4%	1,6-3,2%
Aorta torácica alongada	32	2,2%	1,5-3,0%
Dilatação do tronco da artéria pulmonar	26	1,8%	1,1-2,5%
Aneurisma	12	0,8%	0,4-1,3%
Tromboembolia pulmonar	10	0,7%	0,3-1,1%
Outros achados	24	1,7%	1,0-2,3%

* Busca ativa em pacientes hospitalizados com suspeita clínica. p < 0,0001; Teste qui-quadrado de aderência. COVID-19: doença do coronavírus 2019 (*coronavirus disease* 2019). Fonte: Sistema eletrônico do setor de radiologia.

**Figura 1:** Achados cardiovasculares incidentais em tomografia computadorizada de tórax. Calcificação da parede aórtica e coronária.

**Figura 2:** Achados cardiovasculares incidentais em tomografia computadorizada de tórax. Calcificação da parede aórtica e cardiomegalia.

**Tabela 6:** Achados cardiovasculares incidentais em tomografias computadorizadas de tórax de pacientes com mais de 30 anos e suspeitos de COVID-19, Hospital de Clínicas Gaspar Vianna, março a julho de 2020.

**Achados cardíacos em adultos**	**Frequência**	**% (n = 1.299)**	**Intervalo de confiança**
Calcificação da parede aórtica*	210	16,2%	13,7-28,7%
Cardiomegalia	139	10,7%	8,2-13,2%
Calcificação coronária	58	4,5%	2,0-7,0%
Derrame pericárdio	44	3,4%	0,9-5,9%
Raiz aórtica > 3 cm	31	2,4%	0,0-4,9%
Aorta torácica alongada	23	1,8%	0,0-4,3%
Dilatação do tronco da artéria pulmonar	16	1,2%	0,0-3,7%
Aneurisma	2	0,2%	0,0-2,7%

* p < 0,0001; teste qui-quadrado de aderência.

COVID-19: doença do coronavírus 2019 (*coronavirus disease* 2019). Fonte: Sistema eletrônico do setor de radiologia.

Em relação às afecções aórticas, houve 12 diagnósticos incidentais de aneurisma de aorta torácica (0,83%), sendo sete na aorta ascendente e cinco na aorta torácica descendente. Os 12 aneurismas acometeram 10 pacientes (dois do sexo masculino apresentaram dois aneurismas simultaneamente nas aorta ascendente e descendente).

O exame contrastado foi realizado em 41 pacientes (2,8% do total de exames), e favoreceu diagnósticos alterantivos de dissecção aórtica em 7,3% (três em 41 exames) e de aneurisma da aorta torácica em 9,7% (quatro em 41 exames). Houve 2,4% de achados de úlceras da aorta (dois em 41 exames). As Figuras 3 e 4 ilustram exemplos de afeccões aórticas incidentais evidenciadas em TCs de tórax.

**Figura 3:** Achados cardiovasculares incidentais em tomografia computadorizada de tórax contrastada. Aneurisma da aorta torácica descendente.

**Figura 4:** Achados cardiovasculares incidentais em tomografia computadorizada de tórax contrastada. Dissecção da aorta torácica descendente.

A prevalência de tromboembolismo pulmonar (TEP) em angiotomografias de tórax foi de 24,4% (10 em 41 exames contrastados). Dos exames, 28 foram realizados em pacientes já hospitalizados e 13 em pacientes do pronto atendimento. O achado de TEP ocorreu em dois casos do pronto atendimento e em oito entre os hospitalizados.

## DISCUSSÃO

O expressivo papel do diagnóstico por imagem levou a um aumento no número de exames de TC realizados e, juntamente com sua maior utilização, os radiologistas passaram a observar uma onda de achados não relacionados à indicação clínica específica, os chamados achados incidentais^1^^,^^3^^,^^4^.

O diagnóstico confirmatório de COVID-19 é baseado no RT-PCR em amostras nasais ou da região de orofaringe coletadas com um swab. Pacientes infectados pelo vírus SARS-Cov2 podem apresentar alterações na TC de tórax típicas da doença, como opacidades em vidro fosco. Porém, de acordo com as recomendações do Colégio Brasileiro de Radiologia, o exame deve ser realizado apenas em pacientes sintomáticos e hospitalizados^11^.

Achados incidentais são um fenômeno bem conhecido e comum em exames de imagem^1^^,^^4^^,^^7^^,^^12^. Podem ser classificados, de acordo com sua importância clínica, em maiores, moderados e menores^12^. Os achados maiores incluem lesões suspeitas de doenças malignas, como nódulos da tireoide, alterações da espessura da parede intestinal e massas pancreáticas ou renais sólidas^12^. Achados moderados, isto é, possíveis achados clinicamente relevantes, compreendem cálculos biliares, coleção de fluido pleural ou derrame pleural. Pequenos achados ou achados sem relevância clínica incluem cistos renais ou hepáticos simples, alterações degenerativas na coluna vertebral e calcificação de vasos. Muitos achados incidentais são clinicamente não significativos^1^^,^^2^^,^^4^^,^^7^.

A presente pesquisa evidenciou predomínio de solicitações envolvendo pacientes do sexo feminino e que buscaram assistência no pronto atendimento da instituição, fonte de 48,8% das requisições de exames.

No HCGV, o protocolo usual é o de TC de tórax com espessura de corte de 1,0 mm, baixa dose de radiação e sem contraste endovenoso A grande maioria das TCs de tórax (97,2%) foi realizada sem contraste, sendo reservado o uso de contraste nas particularidades de suspeita de TEP ou de outras afecções vasculares que demandassem melhor análise.

Quanto à origem das solicitações de TCs de tórax, a maioria teve origem no serviço de pronto atendimento (48,8%). Essa expressiva demanda de pacientes externos, em detrimento dos internados em enfermaria ou unidade de terapia intensiva, deve-se provavelmente às características do serviço, que conta com uma urgência porta aberta para pacientes cardiovasculares e nefrológicos.

A TC, muito embora não seja indicada como exame de escolha isolado para o diagnóstico pelas diversas sociedades de especialidade, tornou-se valiosa ferramenta de auxílio diagnóstico nesses pacientes, além de ser útil no acompanhamento da evolução e na detecção de possíveis complicações^12^^,^^13^. Assim, 56% das TCs de tórax solicitadas foram compatíveis com afecção pulmonar pela COVID-19. Os achados mais característicos foram opacidade em vidro fosco e consolidação; no entanto, diversos outros achados podem sugerir afecção pulmonar, dependendo da fase e do tempo de evolução da doença^13^.

Entre as TCs que foram solicitadas para diagnóstico presuntivo de COVID-19, 63,2% tiveram achados incidentais, e a maioria deles consistiram em nódulos pulmonares (15,8%), espessamentos peribrônquicos (13,1%), faixas fibroatelectásicas (11,4%) e atelectasia (9,5%). Entre os achados pulmonares, os nódulos apresentaram significância estatística de p < 0,0001 entre os demais. Nódulos pequenos, acredita-se, não apresentam relevância clínica; no entanto, oito pacientes apresentaram achados de massa pulmonar superior a 3 cm (0,5% da amostra). Isso reforça a hipótese de que a grande maioria dos achados incidentais são irrelevantes; porém, o método também permite detectar lesões assintomáticas que necessitam de melhor gerenciamento^11^. Assim, embora opacidade em vidro fosco bilateral e consolidação tenham sido relatadas como as características predominantes dos exames de imagem por COVID-19, as manifestações da TC no tórax podem variar em diferentes pacientes e estágios^11^.

Achados cardiovasculares incidentais foram evidentes em 51,2% (IC 48,7-53,8%) dos exames tomográficos realizados, e mais de 40% apresentaram mais de um achado. Calcificação das paredes aórtica e coronária, assim como cardiomegalia, foram os mais evidentes em laudos radiológicos. Achados menos comuns envolveram derrame pericárdico e dilatação da raiz aórtica. Esses achados podem ser justificados devido ao perfil epidemiológico apresentado no estudo, no qual aproximadamente 30% dos sujeitos pesquisados têm mais de 60 anos, além do próprio perfil dos pacientes atendidos no hospital, que possui foco cardiovascular e nefrológico.

Na tentativa de homogeneizar o grupo, foi realizada a análise em subgrupo, com a exclusão de 145 exames de indivíduos com menos de 30 anos, perfazendo um total de 1.299 TCs e evidenciando distribuição similar, com predomínio de calcificação coronária e da parede aórtica, cardiomegalia e derrame pericárdico. Portanto, a detecção precoce, ainda que incidental, de doenças com potencial clínico relevante, como calcificação coronária, aneurismas ou dissecções aórticas, pode mudar o prognóstico nessa população e impactar positivamente na redução da mortalidade e no aumento da qualidade de vida.

Surov et al.^2^ mostraram que os achados cardiovasculares podem ser identificados em 6,8% dos pacientes com doenças malignas investigadas por TC. Por outro lado, Jacobs et al.^4^ relataram em seu estudo que a frequência de aneurisma aórtico variou de 0,07% a 3,4%, enquanto que a frequência de dissecção aórtica variou de 0,06% a 0,2%^4^.

Embora os achados cardíacos incidentais possam não ser relevantes para o manejo clínico imediato de pacientes com suspeita de afecção pulmonar pela COVID-19, eles podem influenciar o manejo clínico a longo prazo e favorecer melhores prognósticos. Por exemplo, calcificações coronárias e da parede aórtica são sabidamente marcadoras de aterosclerose e sinais subjacentes de doença cardiovascular, muitas vezes subclínica, podendo influenciar na prevenção primária de eventos ateroscleróticos^14^^-^17. Por outro lado, a cardiomegalia representa uma característica tardia da disfunção ventricular esquerda e da insuficiência cardíaca, ambas com prognóstico desfavorável, podendo desencadear mais investigações e tratamento cardíaco mais efetivo^14^.

Vários estudos têm mostrado que placas ateroscleróticas, calcificações e irregularidades da parede aórtica são muito prevalentes em pacientes com doença cardiovascular^15^^-^17. Outros demonstraram que a calcificação da aorta descendente está relacionada à calcificação das artérias coronárias, importante preditor de doença cardiovascular. No entanto, até o momento, não há estudo de acompanhamento de investigações do valor prognóstico dessas anormalidades^16^^,^^17^.

Sverzellati et al.^18^ relataram que 50% dos 286 exames de TC realizados para fibrose pulmonar, suspeita de embolia pulmonar ou estadiamento do câncer de pulmão tiveram achados cardiovasculares potencialmente significativos. Da mesma forma, Choy et al.^19^ demonstram que 61% dos exames consecutivos de TC de tórax de rotina apresentaram achados cardíacos relatáveis.

Estudos prévios sobre COVID-19 apontam que 63 a 67% dos pacientes que evoluíram a óbito sofriam de comorbidades cardiovasculares, mais comumente hipertensão, diabetes e doença cardíaca coronária^20^^-^22, fatores estes que podem estar relacionados a doenças vasculares^19^^-^22.

Asim, as afecções aórticas foram detectadas incidentalmente em TC de tórax em até 2,4% dos casos, sendo aneurisma a anormalidade mais prevalente, especialmente com a TC sem contraste. Na ausência do uso de contraste intravenoso, dissecções aórticas e ulcerações significativas geralmente são indetectáveis, podendo caracterizar um fator limitante do método^4^. No presente estudo, apenas 2,8% dos exames tomográficos foram realizados com uso de contraste. Aneurismas e dissecções foram observadas respectivamente em 9,7% e 7,3% dos exames contrastados e sugeridas pelos radiologistas em laudo de 1,2% dos exames sem uso de contraste endovenoso. Por outro lado, TEP foi observado em 24,4% dos pacientes que realizaram exame contrastado, representando uma significativa amostra de casos internados em unidade de terapia intensiva, onde predominam os casos de maior gravidade de COVID-19.

Assim, é importante salientar que os achados incidentais configuram-se como um evento importante para o desfecho clínico dos pacientes, visto que boa parte destes possuem comorbidades concomitantes à infecção pelo novo coronavírus e, por conseguinte, tornam-se mais suscetíveis a complicações por essa doença. O diagnóstico incidental, se realizado precocemente e tiver característica relevante, proporciona maiores chances de gerenciamento e tratamento oportuno, e também, por conseguinte, melhores prognósticos à população infectada.

A presente pesquisa apresenta limitações, pois trata-se de estudo retrospectivo e com viés de seleção, visto que analisou uma amostra de pacientes ambulatoriais e hospitalizados em um serviço de referência cardiovascular e em um período de coleta de dados relativamente curto. As TCs foram avaliadas por três radiologistas e não houve, na amostra coletada, caracterização racial ou análise de subgrupos. Alguns achados cardiovasculares foram evidenciados em exames contrastados, como úlceras e dissecções, o que pode limitar exames tomográficos não contrastados.

Não foi realizada a correlação entre os achados cardiovasculares e fatores de risco, pois a pesquisa foi baseada em laudos e exames tomográficos cuja indicação fosse diagnóstico sugestivo de COVID-19. Estudos futuros estão sendo conduzidos na instituição e consideram, na aplicação do protocolo, uma melhor estratificação entre casos ambulatoriais e internados, para resultados mais fidedignos e esclarecedores.

## CONCLUSÕES

Achados cardiovasculares incidentais ocorreram em aproximadamente metade das TCs de tórax de pacientes com suspeita de COVID-19, mais especificamente, calcificações de aorta, cardiomegalia e calcificação coronária.
